# Cetuximab as third‐line rechallenge plus either irinotecan or avelumab is an effective treatment in metastatic colorectal cancer patients with baseline plasma *RAS/BRAF* wild‐type circulating tumor DNA: Individual patient data pooled analysis of CRICKET and CAVE trials

**DOI:** 10.1002/cam4.5699

**Published:** 2023-03-07

**Authors:** G. Martini, D. Ciardiello, V. Famiglietti, D. Rossini, C. Antoniotti, T. Troiani, S. Napolitano, L. Esposito, T. P. Latiano, E. Maiello, M. Del Re, S. Lonardi, G. Aprile, D. Santini, G. Masi, A. Avallone, N. Normanno, F. Pietrantonio, C. Pinto, F. Ciardiello, C. Cremolini, E. Martinelli

**Affiliations:** ^1^ Medical Oncology, Department of Precision Medicine Università degli Studi della Campania “L. Vanvitelli” Naples Italy; ^2^ Division of Gastrointestinal Medical Oncology and Neuroendocrine Tumors IEO, European Institute of Oncology IRCCS Milan Italy; ^3^ Medical Oncology Azienda Ospedaliero‐Universitaria, Department of Translational Research and New Technology in Medicine and Surgery, University of Pisa Pisa Italy; ^4^ Oncologia Medica, Ospedale Casa Sollievo della Sofferenza—IRCCS foundation San Giovanni Rotondo Italy; ^5^ Medical Oncology Veneto Institute of Oncology IOV—IRCCS Padova Italy; ^6^ Department of Oncology San Bortolo General Hospital Vicenza Italy; ^7^ Oncologia Medica Univeristà La Sapienza‐Polo Pontino Rome Italy; ^8^ Oncologia Medica Istituto Nazionale per lo Studio e la Cura dei Tumori “Fondazione Giovanni Pascale” – IRCCS Naples Italy; ^9^ Biologia Cellulare e Bioterapie Istituto Nazionale per lo Studio e la Cura dei Tumori “Fondazione Giovanni Pascale” – IRCCS Naples Italy; ^10^ Medical Oncology Department Fondazione IRCCS Istituto Nazionale dei Tumori Milan Italy; ^11^ Medical Oncology Comprehensive Cancer Centre, AUSL‐IRCCS di Reggio Emilia Reggio Emilia Italy

**Keywords:** cetuximab, immunotherapy, metastatic colorectal cancer, rechallenge

## Abstract

The rechallenge strategy is based on the concept that a subset of patients with *RAS* wild‐type (WT) metastatic colorectal cancer (mCRC) could still benefit of epidermal growth factor receptor (EGFR) inhibition, after progression to an anti‐EGFR based‐therapy. We performed a pooled analysis of two‐phase II prospective trials to determine the role of rechallenge in third‐line mCRC patients with *RAS/BRAF* WT baseline circulating tumor DNA (ctDNA). Individual data of 33 and 13 patients from CAVE and CRICKET trials that received as third‐line therapy cetuximab rechallenge were collected. Overall survival (OS), Progression‐free survival (PFS), Overall response rate (ORR), Stable disease (SD) >6 months were calculated. Adverse events were reported. For the whole 46 patient population, median PFS (mPFS) was 3.9 months (95% Confidence Interval, CI 3.0–4.9) with median OS (mOS) of 16.9 months (95% CI 11.7–22.1). For CRICKET patients, mPFS was 3.9 months (95% CI 1.7–6.2); mOS was 13.1 months (95% CI 7.3–18.9) with OS rates at 12, 18, and 24 months of 62%, 23%, and 0%, respectively. For CAVE patients, mPFS was 4.1 months (95% CI 3.0–5.2); mOS was 18.6 months (95% CI 11.7–25.4) with OS rates at 12, 18, 24 months of 61%, 52%, 21%, respectively. Skin rash was more frequently reported in CAVE trial (87.9% vs. 30.8%; *p* = 0.001), whereas a increased incidence of hematological toxicities was observed in CRICKET trial (53.8%% vs. 12.1%; *p* = 0.003). Third‐line cetuximab rechallenge in combination with either irinotecan or avelumab in *RAS/BRAF* WT ctDNA mCRC patients represents a promising therapy.

## Graphical abstract

Liquid biopsy guides anti‐EGFR rechallenge therapy in CRICKET and CAVE trails.

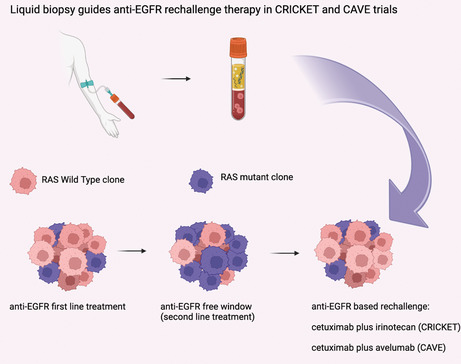

**Novelty and Impact Statements**

Analysis of CRICKET and CAVE phase II trials supports the clinical efficacy of third‐line rechallenge therapy with cetuximab‐based therapies in patients with basal RAS/BRAF WT circulating tumor (ct) DNA. Cetuximab plus irinotecan determines higher response rates with increased occurrence of hematologic toxicities. Cetuximab plus avelumab determines more long‐term stable disease with increased overall survival and skin rash as main toxicity.

**What's new?**

The rechallenge strategy is based on the concept that a subset of patients with *RAS* wild‐type (WT) metastatic colorectal cancer (mCRC) could still take advantage of epidermal growth factor receptor (EGFR) inhibition, after initial progression to an anti‐EGFR based‐treatment.The authors performed a pooled analysis of two‐phase II single arm, prospective trials to determine the role of rechallenge with anti‐EGFR therapies as third‐line treatment in patients with *RAS/BRAF* WT baseline circulating tumor DNA (ctDNA). Individual data of 13 and 33 patients from CRICKET and CAVE trials that received as third‐line therapy cetuximab rechallenge were collected. In the CRICKET, cetuximab was re‐challenged with irinotecan while in CAVE cetuximab was added to avelumab while. The results of the pooled analysis confirmed the promising role of rechallenge with cetuximab‐based regimens (irinotecan and avelumab) in the absence of *RAS/BRAF* mutation at baseline ctDNA analysis, with a mPFS and a mOS of approximately 4 and 17 months, respectively.

## 1 INTRODUCTION

The anti‐epidermal growth factor receptor (EGFR) monoclonal antibodies cetuximab and panitumumab in combination with a chemotherapy backbone, such as FOLFIRI or FOLFOX, represent a standard of care in *RAS* wild type (WT) metastatic colorectal cancer (mCRC).^1^, ^2^, ^3^ Clinical activity of retreatment with anti‐EGFR drugs is recently emerged in a subset of patients with *RAS* WT mCRC, that had previously benefited from anti‐EGFR‐based therapies.^1^, ^4^ This treatment strategy has been defined anti‐EGFR rechallenge and is based on the biological hypothesis of the dynamic evolution of cancer cell clones during treatment.^5^ Approximately 30–50% of mCRC patients develop *RAS* mutant cancer cell clones during anti‐EGFR therapy, which leads to disease progression after initial anti‐tumor response.^2^ These anti‐EGFR‐resistant cancer clones decay over time after the interruption of EGFR inhibitor treatment with half‐life of approximately 4 months.^6^ As a consequence, at this time mCRC patients will have a tumor that is composed of *RAS* WT cancer cells and is again potentially sensitivity to anti‐EGFR blockade.^1^, ^4^

Despite the potential clinical relevance that rechallenge might represent in EGFR‐dependent *RAS* WT mCRC, only a limited number of relatively small size and mostly retrospective anti‐EGFR rechallenge studies has been published so far.^7^, ^8^, ^9^, ^10^ In this respect, CRICKET and CAVE phase II were the first prospective clinical trials that reported clinically meaningful antitumor activity with an acceptable safety profile of rechallenge therapy with cetuximab in combination with the topoisomerase I inhibitor irinotecan or with the anti‐programmed death ligand 1 (PD‐L1) monoclonal antibody avelumab, respectively, in a subset of patients with *RAS* WT mCRC [i.e., with *RAS/BRAF* WT disease at baseline, as assessed by liquid biopsy analysis of circulating tumor DNA, (ctDNA)].^11^, ^12^ Currently, analysis of *RAS/BRAF* WT ctDNA by liquid biopsy represents the only biomarker, that could identify patients that are potentially benefiting from anti‐EGFR rechallenge, although new findings suggest that MAPK mutational status as well might be useful for rechallenge strategy.^11^, ^12^, ^13^, ^14^ In addition, other potential biomarkers of response to anti‐EGFR rechallenge have been proposed such as anti‐EGFR‐induced skin toxicity and pre‐treatment values of neutrophil‐to‐lymphocyte ratio (NLR),^15^, ^16^ but larger prospective studies are needed.

Here we report the results of the pooled analysis with individual patient data of CRICKET and CAVE clinical trials, which share the same inclusion criteria, to better clarify the role of cetuximab‐based rechallenge therapy in the third‐line treatment of mCRC.

## 2 MATERIALS AND METHODS

### 2.1 Study design and participants

Individual patient data (IPD) from two consecutive non‐profit, academic single arm phase II trials (CRICKET and CAVE) were collected.^11^, ^12^ Overall survival (OS), Progression‐free survival (PFS), Overall response rate (ORR), Stable disease (SD) >6 months were calculated. Grade 1–3 adverse events were reported. The CRICKET trial explored the activity of biweekly cetuximab, 500 mg/m^2^, plus irinotecan, 180 mg/m^2^, whereas the CAVE trial evaluated the combination of the anti‐PDL1 avelumab (10 mg/kg every 2 weeks) and cetuximab (400 mg/m^2^ and, subsequently, 250 mg/m^2^ weekly). Both trials were conducted in the rechallenge setting in *RAS* WT mCRC. The main inclusion criteria were identical. In fact, patients should have obtained a major response during first‐line chemotherapy plus an anti‐EGFR drug and should have received a second‐line therapy after progression of disease. The time between the end of first‐line therapy and the start of third‐line therapy should have been at least 4 months. In both trials ctDNA analysis was performed before treatment initiation for an exploratory prospective evaluation. Of note, being two consecutive non‐profit, academic Italian trials, most centers were participating to both CRICKET and CAVE studies. Data were verified for consistency with the original publications; discrepancies were resolved, before collecting the final pooled database.

### 2.2 Outcomes and statistics analysis

χ^2^ test or Fisher's exact test were used as appropriate. Survival curves were estimated with the Kaplan–Meier method. Odds ratios (OR) and 95% confidence intervals (CI) were estimated with the logistic regression model, hazard ratios (HR), and 95% CI were estimated with the Cox proportional hazard model. All statistic tests were two‐sided, and P values ≤0.05 were considered significant. Univariate analysis identified prognostic factors for PFS and OS. Multivariate analyses for PFS and OS were then performed, retaining factors at a *p* < 0.10 level from the univariate model. Patient data were collected at data cut‐off of February 28, 2019 for CRICKET and June 30, 2022 for CAVE. Statistical analyses were performed using the SPSS package (version 23, IBM).

## 3 RESULTS

From the initial population of 28 (CRICKET) and 77 (CAVE) patients, 13 and 33 patients with baseline plasma *RAS/BRAF* WT ctDNA, that received as third‐line cetuximab rechallenge therapy (which was combined with irinotecan in CRICKET and with avelumab in CAVE, respectively), were analyzed. (Figure S1). For CRICKET trial, the median follow‐up was 27.4 months; the PFS events were 13/13 (100%); the OS events were 13/13 (100%). For CAVE trial, the median follow‐up was 27.2 months; the PFS events were 33/33 (100%); the OS events were 26/33 (78.8%) with seven patients alive at the time of the analysis. In the CRICKET cohort, median age was 68.5; 76.9% of patients had Eastern Cooperative Oncology Group (ECOG) performance status (PS) of 0. In CAVE, median age was 60; 69.7%. of patients had ECOG‐PS of 0. Among the two groups of patients, no difference was observed according to number of the metastatic sites and tumor location. A higher proportion of CRICKET patients received surgical removal of the primary tumor. Patient characteristics are summarized in Table 1.

**TABLE 1:** Patient characteristics.

		Pooled, *N* = 46, *n* (%)	CRICKET, *N* = 13, *n* (%)	CAVE, *N* = 33, *n* (%)	*p*‐value
Age, median (range)		60 (30–74)	68.5 (45–77)	60 (30–74)	*p* = 0.035
Gender	Male	27 (58.7)	9 (69.2)	18 (54.4)	*p* = 0.36
	Female	19 (41.3)	4 (30.8)	15 (45.5)	
ECOG	0	33 (71.7)	10 (76.9)	23 (69.7)	*p* = 0.18
	1	12 (26.1)	2 (15.4)	10 (30.3)	
	2	1 (2.2)	1 (7.7)	0	
Number of metastatic sites	≤ 2	33 (71.7)	8 (61.5)	25 (75.8)	*p* = 0.33
	> 2	13 (28.3)	5 (38.5)	8 (24.2)	
Surgery of primary tumor	Yes	33 (71.7)	12 (92.3)	21 (63.4)	*p* = 0.073
	No	13 (28.3)	1 (7.7)	12 (36.4)	
Primary tumor location	Right‐sided	4 (8.7)	3 (23.1)	1 (3)	*p* = 0.062
	Left‐sided	42 (91.3)	10 (76.9)	31 (97)	
Neutrophil lymphocyte ratio	≥3	20 (43.5)	6 (46.2)	14 (42.4)	*p* = 0.82
	<3	26 (56.5)	7 (53.8)	19 (57.6)	

**FIGURE 1:**
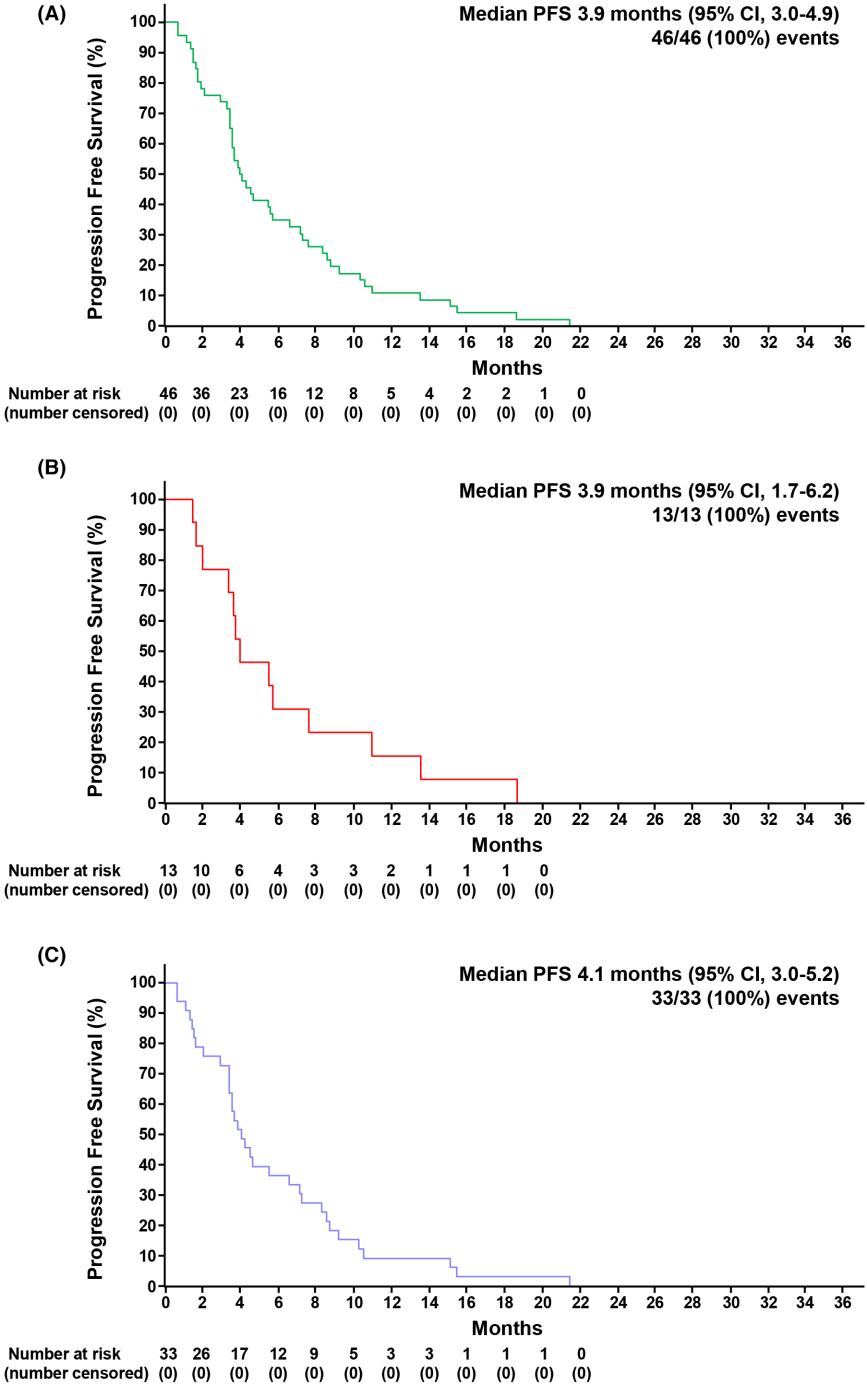
(A) Kaplan–Meier estimates of pooled progression‐free survival (PFS); (B) Kaplan–Meier estimates of PFS (CRICKET); (C) Kaplan–Meier estimates of PFS (CAVE).

**FIGURE 2:**
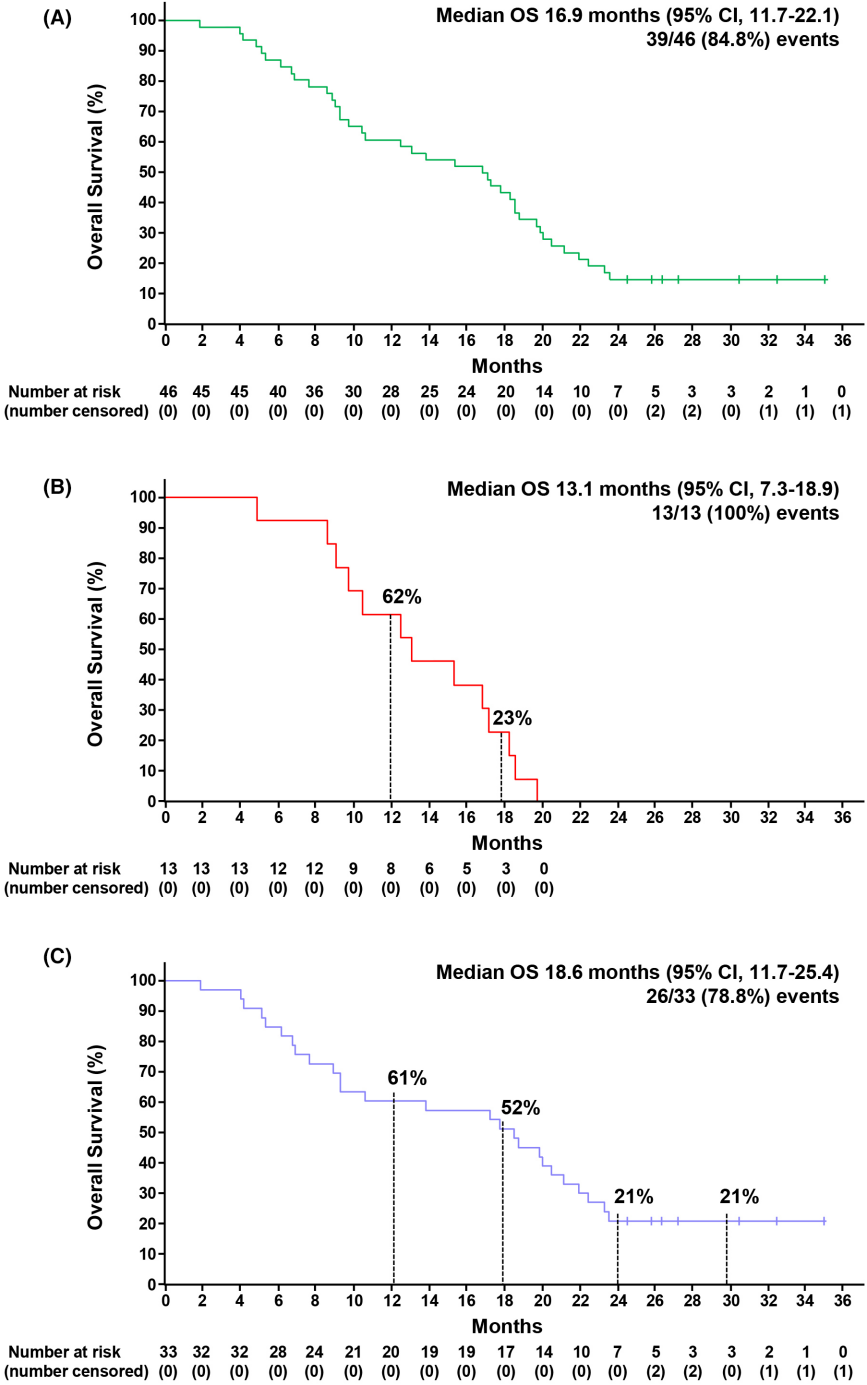
(A) Kaplan–Meier estimates of pooled overall survival (OS); (B) Kaplan–Meier estimates of OS (CRICKET); (C) Kaplan–Meier estimates of OS (CAVE).

After pooling the data (for 46 patients), median PFS (mPFS) was 3.9 months (95% CI 3.0–4.9) (Figure 1A). Median PFS in CRICKET was 3.9 months (95% CI 1.7–6.2) (Figure 1B), whereas it was 4.1 months (95% CI 3.0–5.2) in CAVE (Figure 1C). Pooled median OS (mOS) was 16.9 months (95% CI 11.7–22.1) (Figure 2A). In CRICKET, mOS was 13.1 months (95% CI 7.3–18.9), with OS rates at 12, 18 and 24 months of 62%, 23%, and 0% (Figure 2B). In CAVE, mOS was 18.6 months (95% CI 11.7–25.4) with OS rates at 12, 18, 24, 30, and 35 months of 61%, 52%, 21%, 21%, and 21%, respectively (Figure 2C). The overall response rate (ORR) and SD >6 months were 38.5% (95% CI 13.9–58.4) and 7.7% (95% CI 0.2–36), respectively, in the CRICKET trial. In the CAVE trial, ORR and SD >6 months were 9.1% (95% CI 1.9–24.3) and 30.3% (95% CI, 15.6–48.7), respectively. By multivariate analysis, only neutrophil/lymphocyte ratio ≥3 was a significant predictor of worse OS (Hazard Ratio, HR, 2.25; 95% CI 1.11–4.51; *p* = 0.023) (Figure S2).

After disease progression, a subsequent line of therapy was administered to 84.6% of patients in CRICKET and to 81.8% of patients in CAVE. Regorafenib was administered to 53.8% of CRICKET patients and to 30.3% of CAVE patients, whereas 15.4% of CRICKET patients and 27.3% of CAVE patients were treated with trifluridine‐tipiracil. Subsequent line details are listed in Table S2.

The incidence of skin toxicities was higher in CAVE trial (87.9% vs. 30.8% patients, *p* = 0.001). Globally, non‐hematological toxicities were more often reported in CAVE trial (87.9% vs. 30.8%; *p* = 0.001). However, a few non‐hematological toxicities, such as diarrhea, hand‐foot syndrome, nausea, and stomatitis, were more frequently associated to the CRICKET trial (15.4% vs. 0%; *p* = 0.075). Finally, hematological toxicities (anemia, neutropenia, febrile neutropenia, decreased platelet count), that more frequently led to treatment delay or dose reduction, were prevalent in CRICKET trial (53.8 vs. 12.1%, *p* = 0.003) (Table S1).

## 4 DISCUSSION

Thanks to an improvement in the continuum of care, the OS of patients with *RAS* WT mCRC is progressively increased over the last decade and now reaches 35–40 months.^1^, ^2^ Interestingly, emerging evidence suggest that patients with *RAS* WT mCRC could advantage of EGFR blockade even after initial progression.^1^, ^4^ Without the selective pressure of anti‐EGFR mAbs there is a progressive decay of *RAS/BRAF* mutant resistant clones, supporting the rechallenge with cetuximab/panitumumab‐based treatments. However, only few data derived from retrospective and no‐randomized study are currently available. So far, the identification of the optimal type of rechallenge strategy (anti‐EGFR single agent, combination with chemotherapy or with immunotherapy) and the evaluation of potential biomarkers is still under investigation.

In this scenario, waiting for the results of randomized studies, we performed and individualized data pooled analysis of the CAVE and CRICKET studies that represented the first prospective trials assessing the role of retreatment with EGFR inhibitors in refractory patients with mCRC.^11^, ^12^

Of note, cetuximab was combined with two drugs based on different pre‐clinical/clinical rationale^17^, ^18^ . In the BOND study the addition of cetuximab to irinotecan was able to restore sensibility to the drugs in irinotecan‐refractory patients with mCRC.^17^

As demonstrated in preclinical and translational model of non‐small cell lung cancer (NSCLC), the addition of avelumab to cetuximab could enhance the activation of natural killer (NK) cells, thus increasing antibody‐dependent cell‐mediated cytotoxicity (ADCC), activating the interaction of NK with dendritic cells, and enhancing major histocompatibility complex (MHC) class II molecule expression, which could result in more effective cetuximab‐induced cancer cell death.^18^

The results of the pooled analysis confirmed the promising role of rechallenge with cetuximab‐based regimens (irinotecan and avelumab) in the absence of resistant mutation at baseline ctDNA evaluation, with a mPFS and a mOS of approximately 4 and 17 months, respectively. These data are intriguing in the context of medical management of pretreated mCRC patients, since the only two currently approved therapies for this indication (regorafenib and trifluridine/tipiracil) are mainly cytostatic with 2–3 months mPFS and 7–9 months mOS.^19^, ^20^, ^21^ While the two regimes were both active, some difference in the efficacy and safety profile were observed. The reintroduction of a chemotherapy drug as irinotecan in addition to cetuximab lead to a higher tumor shrinkage with a higher ORR 38.5%. However, responses were not durable. On the other hand, immune‐rechallenge with cetuximab plus avelumab, showed a lower response rate, with a subset of patients that exhibited a long‐term disease control (SD >6 months in 30.8% of patients) and survival (mOS, 18.6 months with 21% of patients alive up to 35 months). In a later line setting the prolongation of the quantity of life with a good treatment tolerability is a primary goal. In this regard, the CAVE and CRICKET study the rechallenge therapy showed an acceptable safety profile. Skin rash was more frequent when cetuximab was combined with avelumab. A higher incidence of hematological and gastrointestinal toxicities (diarrhea, nausea, and stomatitis) was observed when cetuximab is combined with chemotherapy. In both studies most of the patients maintained a good performance status that permitted to receive a subsequent line of treatment. Several studies are currently on‐going evaluating the role of rechallenge strategies.^4^

Recently an interim analysis of a phase I study (AVETUXIRI trial, NCT03608046) investigating the combination of cetuximab, irinotecan, and avelumab was presented.^22^, ^23^

The triplet combination displayed an feasible safety profile, most relevant grade 3–4 adverse event was diarrhea with a complete recovery after dose reduction or interruption. Of note, in the *RAS* wt population three patients experienced a response (ORR 30%, 3/10) with a 6‐months PFS and 12‐months OS rates were respectively 40.0% and 50.0%. Based on these preliminary results the study is currently on‐going, mature date will be presented.

The current study has different limitations: (a) first the small population render these results as hypothesis generating; (b) second, while the inclusion criteria of the two studies and the patients' characteristics were similar, there is an imbalance in the number of patients treated with cetuximab plus avelumab (*N* = 33) compared with cetuximab plus irinotecan (*N* = 13). Despite these limitations, results of IPD from CAVE and CRICKET studies proves that third‐line therapeutic rechallenge with cetuximab plus either irinotecan or avelumab is a promising treatment for mCRC patients with baseline plasma *RAS/BRAF* WT ctDNA. Since these results derive from subgroup analysis of two single arm phase II trials, larger prospective studies are needed.

To this aim, the CAPRI‐2 GOIM trial (EudractCT number: 2020–003008‐15; NCT05312398), a phase II study, in which treatment of *RAS/BRAF WT* mCRC patients is driven by the results of liquid biopsy‐assessed plasma *RAS/BRAF* ctDNA status throughout three lines of therapy, is currently enrolling patients. In this trial, patients with *RAS/BRAF* WT ctDNA at the time of third‐line treatment will be treated with cetuximab plus irinotecan. Further, a randomized phase II study, which compares cetuximab single agent or in combination with avelumab as rechallenge therapy in mCRC patients with plasma *RAS/BRAF* WT ctDNA, has been recently started (CAVE‐2 GOIM trial, EudractCT number: 2021–004593‐56; NCT05291156).^24^

## AUTHOR CONTRIBUTIONS

**Giulia Martini:** Conceptualization (lead); data curation (lead); formal analysis (equal); methodology (lead); supervision (lead); validation (lead); writing – original draft (equal); writing – review and editing (lead). **Davide Ciardiello:** Conceptualization (lead); data curation (lead); formal analysis (equal); methodology (lead); supervision (lead); validation (lead); writing – review and editing (lead). **Vincenzo Famiglietti:** Methodology (equal); project administration (equal); software (equal). **Daniele Rossini:** Data curation (equal); investigation (equal); resources (equal); writing – review and editing (equal). **Carlotta Antoniotti:** Data curation (equal); investigation (equal); resources (equal); writing – review and editing (equal). **Teresa Troiani:** Conceptualization (equal); investigation (equal); resources (equal); writing – review and editing (equal). **Stefania Napoletano:** Data curation (equal); investigation (equal); resources (equal); writing – review and editing (equal). **Lucia Esposito:** Methodology (equal); resources (equal); supervision (equal). **Tiziana Pia Latiano:** Data curation (equal); investigation (equal); resources (equal); writing – review and editing (equal). **Evaristo Maiello:** Data curation (equal); investigation (equal); resources (equal); writing – review and editing (equal). **Marzia Del re:** Data curation (equal); investigation (equal); resources (equal); writing – review and editing (equal). **Sara Lonardi:** Data curation (equal); methodology (equal); resources (equal); writing – review and editing (equal). **Giuseppe Aprile:** Data curation (equal); methodology (equal); resources (equal); writing – review and editing (equal). **Daniele Santini:** Conceptualization (equal); methodology (equal); resources (equal); writing – review and editing (equal). **Gianluca Masi:** Data curation (equal); methodology (equal); resources (equal); writing – review and editing (equal). **Antonio Avallone:** Data curation (equal); methodology (equal); resources (equal); writing – review and editing (equal). **Nicola Normanno:** Conceptualization (equal); methodology (equal); resources (equal); writing – review and editing (equal). **Filippo Pietrantonio:** Conceptualization (equal); methodology (equal); resources (equal); writing – review and editing (equal). **CARMINE PINTO:** Conceptualization (equal); methodology (equal); resources (equal); writing – review and editing (equal). **Fortunato Ciardiello:** Conceptualization (equal); methodology (equal); resources (equal); writing – original draft (equal); writing – review and editing (equal). **Chiara Cremolini:** Conceptualization (equal); methodology (equal); resources (equal); writing – original draft (equal); writing – review and editing (lead). **erika martinelli:** Conceptualization (lead); data curation (lead); methodology (lead); resources (lead); supervision (lead); validation (lead); writing – original draft (lead); writing – review and editing (lead).

## CONFLICT OF INTEREST STATEMENT

**Giulia Martini**: received honoraria from Servier and Incyte. **Davide Ciardiello** travel support from Sanofi, BMS, and Merck Serono. **Daniele Rossini** received honoraria from Merck Sharp & Dohme (MSD). **Teresa Troiani** has served as advisor and speaker for Roche, Merck‐Serono, Sanofi, Servier, Novartis, Bayer. **Tiziana Pia Latiano** has served as speaker for Servier. **Evaristo Maiello** has served as advisor and speaker for Astra Zeneca, Eli Lilly, Servier, Sanofi Genzyme, Roche, Merck, Eisai, Pfizer. **Marzia Del Re** received fees from Astellas, AstraZeneca, Celgene, Novartis, Pfizer, Bio‐Rad, Janssen, Sanofi‐Aventis, Roche, MSD, Lilly and Ipsen; honoraria for advisory role: Astra Zeneca, MSD, Ipsen, Janssen, Sanofi‐Aventis, and Amgen. **Sara Lonardi** has a consulting or an advisory role for Amgen, Merck Serono, Lilly, AstraZeneca, Incyte, Daiichi‐Sankyo, BMS, Servier, and MSD; has received research funding from Amgen, Merck Serono, Bayer, Roche, Lilly, AstraZeneca, and BMS; and has received speakers' fees from Roche, Lilly, BMS, Servier, Merck Serono, Pierre‐Fabre, GlaxoSmithKline, and Amgen. **Giuseppe Aprile** has a consulting or an advisory role for Amgen, Astellas, AstraZeneca, BMS, Lilly, Merck Serono, MSD, Servier Gianluca Masi has served as advisory and speaker for Amgen, Roche , MSD, Eisai, Merck Serono, and Astra Zeneca. **Antonio Avallone** has served as advisor or a speaker for Amgen, MSD, EISAI, AstraZeneca, and Servier. **Nicola Normanno** has served as advisor and speaker for MSD, Qiagen, Biocartis, Incyte, Roche, BMS, MERCK, Thermofisher, Boehringer Ingelheim, Astrazeneca, Sanofi, Eli Lilly, Bayer. **Filippo Pietrantonio** received honoraria from Amgen, Merck Serono, Lilly, Sanofi, Servier, Bayer, MSD, and AstraZeneca, and research grants from Bristol Myers Squibb (BMS) and AstraZeneca, Incyte, and Agenus. **Carmine Pinto** reports outside the submitted work personal fees for advisory role, speaker engagements, and travel and accommodation expenses from Amgen, Astellas, Astra‐Zeneca, Bayer, Bristol Meyer Squibb, Clovis Oncology, Ipsen, Janssen, Incyte, Merck‐Serono, Merck Sharp and Dohme, Novartis, Roche, and Sanofi. **Fortunato Ciardiello** has served as advisor and speaker for Roche, Amgen, Merck‐Serono, Pfizer, Sanofi, Bayer, Servier, BMS, Cellgene, Lilly. Received institutional Research Grants form Bayer, Roche, Merck‐Serono, Amgen, AstraZeneca, Takeda. VF, SN, LE, and GM declare no competing interests. **Chiara Cremolini** has received personal fees from Roche, Amgen, Bayer, Servier, Merck, MSD, Pierre Fabre, Nordic Pharma, and Organon; has received research funding from Merck Serono, Servier, Bayer, and Amgen; and has a consulting or an advisory role with Bayer, Amgen, Servier, and Pierre Fabre. **Erika Martinelli** receipt of honoraria or consultation fees for speaker, consultancy or advisory roles: Amgen, Bayer, Eisai, Merck Serono, Pierre Fabre, Roche, Servier, Incyte, ESMO, travel grant: AstraZeneca, Pierre Fabre. All other authors declare no competing interests.

## ETHICS STATEMENT

The CRICKET study was approved by the Ethic Committee of Azienda Ospedaliera Pisana and is registered with ClinicalTrials.gov Identifier: NCT02296203. The CAVE‐mCRC trial was approved by the Ethic Committee of Università degli Studi della Campania Luigi Vanvitelli, Azienda Ospedaliera Universitaria‐AORN “Ospedali dei Colli”, Napoli, Italy. CAVE‐mCRC trial is registered with Eudract.ema.europa.eu, EudraCT number: 2017–004392‐32 and ClinicalTrial.gov identifier: NCT04561336. The approval of a formal protocol for the IPD analysis was obtained from the principal investigators of the trials. All patients provided written informed consent before entering the trial. The study was conducted in accordance with the principles of the Declaration of Helsinki and the International Conference on Harmonization, and Good Clinical Practice guidelines.

## Supporting information


Figure S1.

Figure S2.

Table S1.

Table S2.

## Data Availability

The data that support the findings of our study and further information are available from the corresponding author upon reasonable request.
