# Supplementary figures and images for: Cetuximab as third‐line rechallenge plus either irinotecan or avelumab is an effective treatment in metastatic colorectal cancer patients with baseline plasma RAS/BRAF wild‐type circulating tumor DNA: Individual patient data pooled analysis of CRICKET and CAVE trials
Source: Cancer Med. 2023 Mar 7;12(8):9392–400. doi: 10.1002/cam4.5699 (PMC10166888; doi:10.1002/cam4.5699)

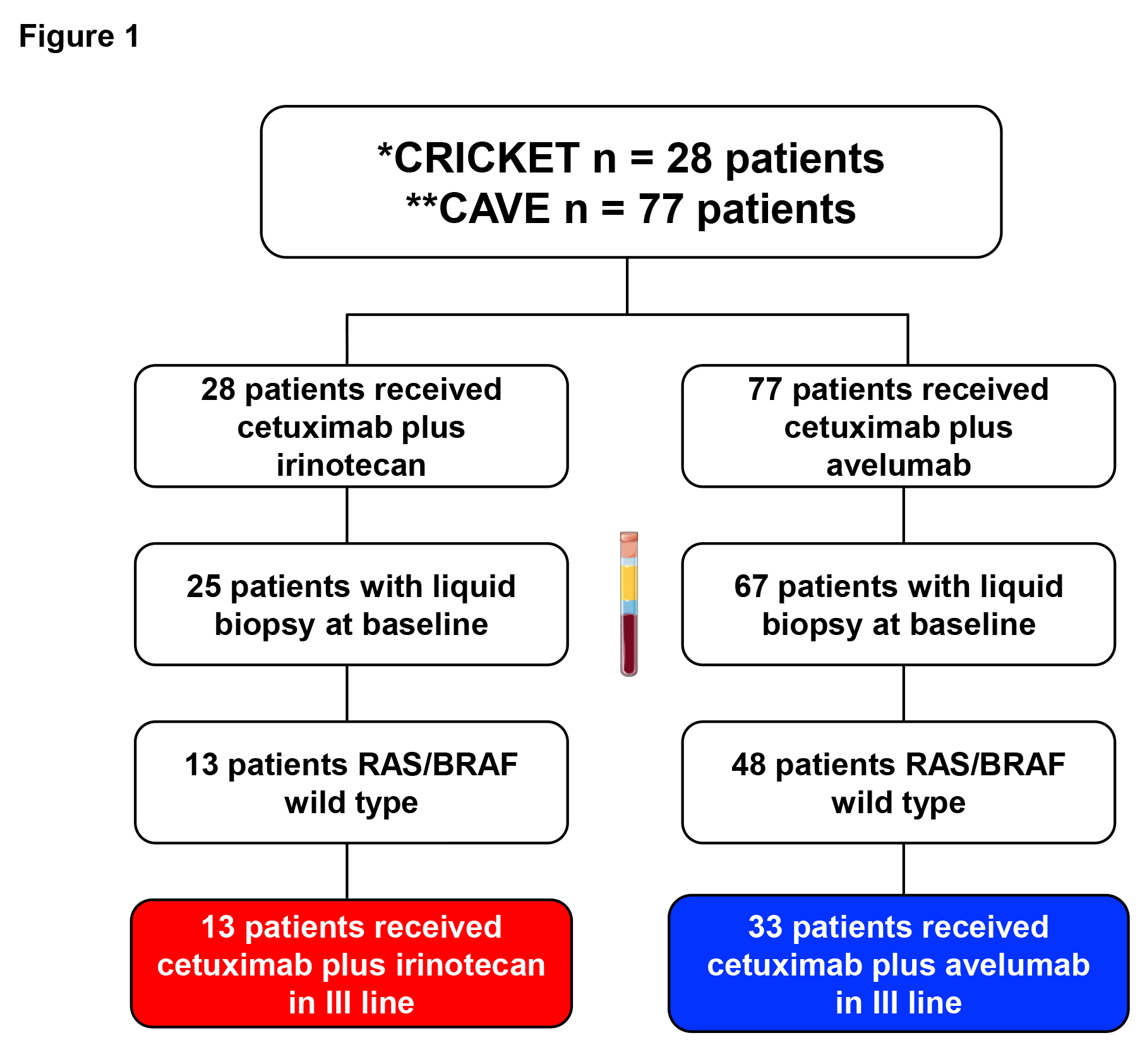

Supplement: Supplementary file 1 — Figure S1. [file CAM4-12-9392-s001.jpeg]

**
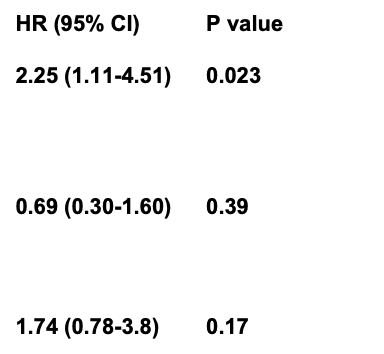

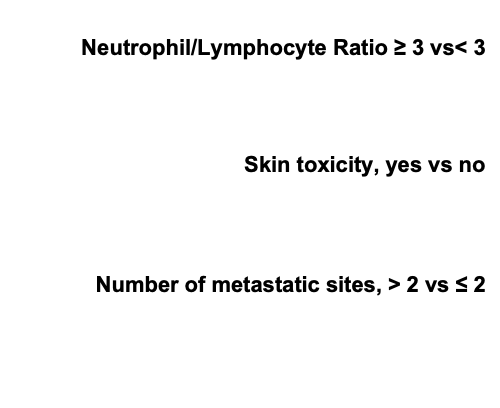

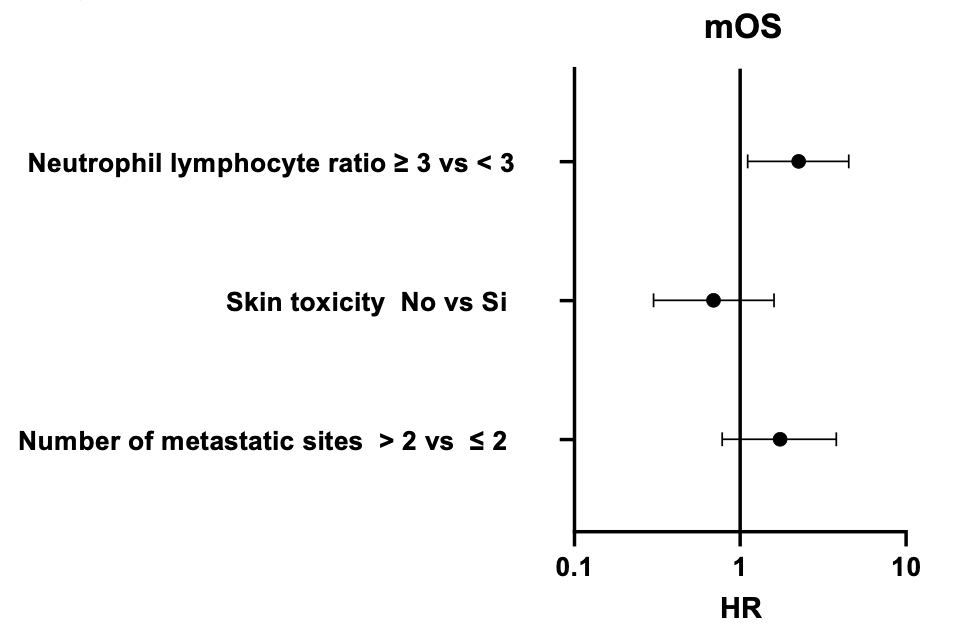
Supplementary figure 1**

Supplement: Supplementary file 2 — Figure S2. [file CAM4-12-9392-s002.docx]
