# Supplementary material for: Cetuximab as third‐line rechallenge plus either irinotecan or avelumab is an effective treatment in metastatic colorectal cancer patients with baseline plasma RAS/BRAF wild‐type circulating tumor DNA: Individual patient data pooled analysis of CRICKET and CAVE trials
Source: Cancer Med. 2023 Mar 7;12(8):9392–400. doi: 10.1002/cam4.5699 (PMC10166888; doi:10.1002/cam4.5699)
Supplement: Supplementary file 3 — Table S1. [file CAM4-12-9392-s003.docx]

**Supplementary table 1**

|  |  | **Pooled**  **N=46**  **n (%)** | **CRICKET**  **N=13**  **n (%)** | **CAVE**  **N=33**  **n (%)** | **P-value** |
| --- | --- | --- | --- | --- | --- |
| **Skin toxicity** | Yes  No | 33 (71.7)  13 (28.3) | 4 (30.8)  9 (69.2) | 29 (87.9)  4 (12.1) | p=0.001 |
| **Haematological toxicity** | Yes  No | 2 (4.3)  44 (95.7) | 2 (15.4)  11 (84.6) | 0 (0)  33 (100) | p=0.075 |
| **Non haematological toxicity** | Yes  No | 11 (23.9)  35 (76.1) | 7 (53.8)  6 (46.2) | 4 (12.1)  29 (87.9) | p=0.003 |
| **Dose reduction** | Yes  No | 9 (19.6)  37 (80.4) | 5 (61.5)  8 (38.8) | 4 (12.1)  29 (87.9) | p=0.043 |
| **Median number of cycles received** |  | 8.5  (1-36) | 9  (1-36) | 8  (1-30) | p=0.28 |

**Non-haematologic toxicities:** diarrhea, hand-foot syndrome, nausea and stomatitis

**Haematologic toxicities:** anaemia, neutropenia and febrile neutropenia, decreased platelet count
