# Supplementary material for: Cetuximab as third‐line rechallenge plus either irinotecan or avelumab is an effective treatment in metastatic colorectal cancer patients with baseline plasma RAS/BRAF wild‐type circulating tumor DNA: Individual patient data pooled analysis of CRICKET and CAVE trials
Source: Cancer Med. 2023 Mar 7;12(8):9392–400. doi: 10.1002/cam4.5699 (PMC10166888; doi:10.1002/cam4.5699)
Supplement: Supplementary file 4 — Table S2. [file CAM4-12-9392-s004.docx]

**Table 2**

|  |  | **Pooled**  **N=46**  **n (%)** | **CRICKET**  **N=13**  **n (%)** | **CAVE**  **N=33**  **n (%)** | **P-value** |
| --- | --- | --- | --- | --- | --- |
| **Subsequent line of therapy** | Yes  No | 38 (82.6)  8 (17.4) | 11(84.6)  2 (15.4) | 27 (81.8)  6 (18.2) | p=0.82 |
| **Post-study treatment:** |  |  |  |  |  |
| **NA** |  | 7 (15.2) | 2 (15.4) | 5 (15.2) |  |
| **Regorafenib** |  | 17 (37) | 7 (53.8) | 10 (30.3) |  |
| **Trifluridine/tipiracil** |  | 11 (23.9) | 2 (15.4) | 9 (27.3) |  |
| **Trifluridine/tipiracil + bevacizumab** |  | 5 (10.9) | 0 (0) | 5 (15.2) |  |
| **Pembrolizumab** |  | 1 (2.2) | 0 (0) | 1 (3.0) |  |
| **Folfiri** |  | 1 (2.2) | 0 (0) | 1 (3.0) |  |
| **Xelox** |  | 1 (2.2) | 0 (0) | 1 (3.0) |  |
| **Capecitabina** |  | 1 (2.2) | 1 (7.7) | 0 (0) |  |
| **Capecitabine+bevacizumab** |  | 1 (2.2) | 1 (7.7) | 0 (0) |  |
| **Cabozantinib** |  | 1 (2) | 0 (0) | 1 (3) |  |
